# Characterization of polar surface groups on siliceous materials by inverse gas chromatography and the enthalpy–entropy compensation effect

**DOI:** 10.3389/fchem.2023.1084046

**Published:** 2023-03-30

**Authors:** Ralf Meyer, Kai Mueller, Sergej Naumov, Frank Bauer, Dirk Enke

**Affiliations:** ^1^ Institute of Chemical Technology, Leipzig University, Leipzig, Germany; ^2^ Leibniz Institute of Surface Engineering, Leipzig, Germany

**Keywords:** inverse gas chromatography, porous silica, surface energy, polar surface groups, acid–base properties, enthalpy–entropy compensation, isokinetic temperature, hydrogen bonds

## Abstract

Surface-modified porous silica is a well-established composite material. To improve its embedding and application behavior, adsorption studies of various probe molecules have been performed using the technique of inverse gas chromatography (IGC). For this purpose, IGC experiments were carried out in the infinite dilution mode on macro-porous micro glass spheres before and after surface modification with (3-mercaptopropyl)trimethoxysilane. To provide information about the polar interactions between probe molecules and the silica surface, in particular, eleven polar molecules have been injected. In summary, the free surface energy for pristine silica ( $\gamma_{S}^{total}$ = 229 mJ/m^2^) and for (3-mercaptopropyl)trimethoxysilane-modified silica ( $\gamma_{S}^{total}$ = 135 mJ/m^2^) indicates a reduced wettability after surface modification. This is due to the reduction of the polar component of the free surface energy ( $\gamma_{S}^{SP}$ ) from 191 mJ/m^2^ to 105 mJ/m^2^. Simultaneously, with the reduction of surface silanol groups caused by surface modification of silica and, therefore, the decrease in polar interactions, a substantial loss of Lewis acidity was observed by various IGC approaches. Experiments with all silica materials have been conducted at temperatures in the range from 90°C to 120°C to determine the thermodynamic parameters, such as adsorption enthalpy ( ${\Delta H}_{ads}$ ) and adsorption entropy ( ${\Delta S}_{ads}$ ), using the Arrhenius regression procedure evaluating the IGC data. With the help of the enthalpy–entropy compensation, two types of adsorption complexes are assumed between polar probe molecules and the silica surface because of different isokinetic temperatures. Identical adsorption complexes with an isokinetic temperature of 370°C have been assigned to alkanes and weakly interacting polar probes such as benzene, toluene, dichloromethane, and chloroform. Polar probe molecules with typical functional groups such as OH, CO, and CN, having the ability to form hydrogen bonds to the silica surface, exhibit a lower isokinetic temperature of 60°C. Quantum chemical calculations of the probe molecules on a non-hydroxylated and hydroxylated silica cluster supported the formation of hydrogen bonds in the case of a strong polar adsorption complex with a bonding distance of 1.7 nm–1.9 nm to the silica surface.

## 1 Introduction

For elucidating catalytic processes and enhancing process efficiency, the characterization of porous catalysts is crucial. While the chemical characterization of the catalyst surface, e.g., by infrared and X-ray photoelectron spectroscopy, is standard practice, the energetic characterization of surface sites is often neglected, although all heterogeneously catalyzed reactions take place at the surface. Therefore, inverse gas chromatography (IGC) has become established as a gas phase method to investigate the adsorption/desorption processes of reactants and products in interactions with particles, granulates, or fibers. This method is able to determine a large number of physicochemical properties, for example, surface energies (Grimsey Ian et al., 2002; Das et al., 2011), acid/base/polar functionality of surfaces (Hamieh et al., 2002), solubility parameters (Adamska and Voelkel, 2006), desorption isotherms (Tisserand et al., 2009), surface heterogeneity (Balard, 1997), and phase transition temperatures (Surana et al., 2003). Although the method was developed in the 1970s, IGC has become popular in recent years. The fundamentals of this method for application to powder materials are summarized by Mohammadi-Jam and Waters (2014), Voelkel et al. (2015), and Williams (2015) in comprehensive reviews.

In general, IGC is a variant of gas chromatography in which the sample material is packed as a column, and interactions with the surface are investigated using organic probe molecules. When injecting particularly small amounts of probe molecules (so-called infinite dilution mode) and, thus, achieving low coverage of the surface, interactions only occur with high-energy adsorption sites and low-energy adsorption sites can be neglected. Ideally, intermolecular interactions between the probes can also be excluded, which results in highly symmetric, Gaussian peak-shaped chromatograms whose retention time remains very accurate even with small changes in sample concentration (Thielmann and Baumgarten, 2000).

The different retention times of individual probe molecules can be used to calculate thermodynamic state variables, such as free energies of the adsorption ( ${∆G}_{ads}$ ), surface free energies ( $\gamma_{S}$ ), and acid–base parameters ( $K_{A}/K_{D}$ ). However, the determination of these parameters showed a significant dependence on the current measurement temperature (Münch and Mertens, 2018; Bauer et al., 2021). Repeating the measurements at different temperatures allows determining temperature-independent parameters, such as adsorption enthalpy ( ${∆H}_{ads}$ ) and adsorption entropy ( ${∆S}_{ads}$ ). Even though the measurements require a considerably higher effort, the importance of these parameters was recently demonstrated by Hamieh et al. (2020) using the example of metal–organic frameworks (MOFs). By taking into account the temperature dependence of the adsorption surfaces of probe molecules, much more accurate results can be obtained.

Adsorption enthalpy and adsorption entropy have an additional mechanistic–diagnostic value. By investigating the correlation of ${∆H}_{ads}$ and ${∆S}_{ads}$ in the form of the so-called enthalpy–entropy compensation, the mechanistic interpretation of thermodynamic and kinetic activation parameters of complex systems can be studied (Starikov and Nordén, 2007).

For example, Ruthven and Kaul (1998) showed a correlation between adsorption enthalpy and entropy for the adsorption of linear paraffins on zeolites, silicalite, silica gel, and commercial catalysts. For a linear enthalpy and entropy relation, the isokinetic temperature (T_β_) can be determined from the slope of the regression line. Typically, it is used to highlight differences in adsorbate–adsorbent interactions and as aids in the search for the elucidation of the reaction mechanisms (Denayer et al., 1998; Bond et al., 2000). Thus, Eder and Lercher (1997) showed that the entropy loss is disproportionally stronger for the medium-pore zeolite H-ZSM-5 compared to the large-pore zeolites H-FAU, H-BEA, and H-MOR. This higher entropy loss observed on MFI suggests more pronounced interactions with the lattice of MFI and tighter bound surface complexes than with FAU.

With respect to the application of IGC, various publications show the remarkable relevance of the correlation between ${∆H}_{ads}$ and ${∆S}_{ads}$ , mainly for dispersive interactions (Tümsek and İnel, 2003; Díaz et al., 2007; Huang, 2007; Yusuf et al., 2016). For a study of the compensation effect on silica surfaces, however, it was important to use polar probes since adsorption complexes can be expected in addition to those induced by van der Waals forces. These specific interactions are mainly electron donor–acceptor interactions, electrostatic interactions, pi bonds, and hydrogen bonds (Kraus et al., 2015).

The primary task of this work was comparing the surface functionalities of macro-porous silica spheres (intended for use in modern sensor technology) before and after extensive modification with (3-mercaptopropyl)trimethoxysilane (MPTMS) used as a cross-linking silane. It is obvious that silica hydrophobization by MPTMS multilayers should really and quickly be confirmed through IGC using the adsorption of a few linear paraffins at any temperature. However, measuring surface energies of complex surfaces with exclusively alkane probes is worthless, as has already been shown (Das et al., 2011; Bauer et al., 2019; Pal et al., 2019). Thus, we carried out all the work of injecting multiple polar and non-polar probes at multiple temperatures following the different IGC approaches (Gutmann and van Oss), the adsorption enthalpy–entropy determination, and DFT calculations for optimized adsorption complexes. However, even by applying the considerable arsenal of IGC techniques, this study cannot be expected to have entirely new and spectacular results on the energetic characterization of the well-known silica surface sites. Our intention was, consequently, to use IGC in an exemplary manner to obtain a more or less extensive surface energy characterization for new, less well-investigated carrier materials overlooking the chemical intuition for silica materials that removing –OH groups from its surface makes it less polar.

## 2 Materials and methods

### 2.1 Materials

Macro-porous glass spheres of particle size fraction 100–200 µm were produced according to a procedure published elsewhere (Enke et al., 2003). In brief, sodium borosilicate glass spheres with a composition of 70 wt% SiO_2_, 23 wt% B_2_O_3_, and 7 wt% Na_2_O were used as starting materials. To initiate phase separation, the glass was thermally treated at 650°C for 24 h. To remove the pure silica shell, the glass spheres were immersed in a sodium hydroxide solution. The extraction of the sodium-rich borate phase was performed at 80°C with hydrochloric acid. Colloidal silica deposits remaining in the pore system after acidic leaching were removed by treatment with sodium hydroxide solution at room temperature. Finally, the resulting porous glass was washed with deionized water and dried at 120°C.

### 2.2 Surface modification

The surface of porous glass spheres was modified for 1 h at room temperature by reaction with 0.05 mmol silane per m^2^ glass surface (surface coverage ∼6 MPTMS species/nm^2^) dissolved in ethanol and acidified by 0.1 M HCl. After solvent evaporation, the modified porous glass samples were dried at 90°C overnight. To reduce the MPTMS molecules, which are physisorbed at the silica surface after the surface modification, the glass beads were washed three times with water and ethanol, filtered, and finally dried at 100°C overnight.

### 2.3 Structural characterization

For scanning electron microscopy, a LEO GEMINI 1530 from Zeiss with an Everhart–Thornley detector (ETD) was used. The samples were attached to the sample carrier using an adhesive carbon foil and then vapor-deposited with gold. Measurements were performed at an accelerating voltage of 10 kV and a working distance of 5 mm. Particle size determination by laser diffraction was carried out on a Cilas 1064 L instrument. Nitrogen sorption and mercury intrusion measurements were performed by using an Autosorb iQ apparatus (Quantachrome) and a Quantachrome PoreMaster porosimeter, respectively. Elemental analyses were accomplished with a vario Max CHN (Elementar Analysensysteme GmbH) instrument. The amount of organics grafted on the modified porous glass was measured by thermogravimetric analysis (Netzsch STA 409) in air with a heating rate of 10 K/min.

### 2.4 Methods of inverse gas chromatography

IGC experiments were performed on a PerkinElmer Clarus 580 GC apparatus equipped with a flame ionization detector and controlled by the IGC software package from Adscientis SARL (Wittelsheim, France). Samples of 100–300 mg were filled into typical stainless-steel GC-packed columns (10 cm length, o.d., 1/4 in.), accomplished with mechanical vibration. Both ends of the columns were plugged with silane-treated glass wool. All the samples were conditioned at 150°C overnight under a helium flow rate of 20 mL/min. The IGC experiments were performed under the same flow rate at various temperatures (30°C–120°C). On each sample, several molecular probe molecules (C_6_-C_10_ *n*-alkanes, dichloromethane, chloroform, diethyl ether, ethanol, acetone, acetonitrile, ethyl acetate, butanone, tetrahydrofuran, benzene, and toluene) were injected at least two times.

#### 2.4.1 Retention volume and dispersive and specific free energy of adsorption

In the IGC method, gaseous probe molecules are injected to study adsorption to the column material. Thereby, a very high dilution (so-called infinite dilution mode) allows intermolecular interactions to be ignored. Raw data from IGC are the different net retention times ( $t_{N}$ ) for the probe molecules, which are determined using methane as a dead time marker. The retention volume ( $V_{N}$ ) (Mohammadi-Jam and Waters, 2014), as the amount of carrier gas required to purge one probe molecule from the column, can be calculated from the following equation:

$$V_{N}=\dot{V}∙j∙t_{N},$$ (1)

where $\dot{V}$ is the carrier gas flow and j is the James–Martin correction factor (James and Martin, 1952). For better comparability, the net retention time $V_{N}$ is referenced to the sample mass m and the experimental temperature $T_{m}$ in K . This results in the specific retention volume $V_{G}$ according to the following equation:

$$V_{G}=V_{N}∙\frac{273.15\;K}{m∙T_{m}}.$$ (2)

More details of the theoretical background can be found in recent IGC publications (Gholami et al., 2020; Hamieh et al., 2020).

Thermodynamic calculations (Schultz et al., 1987) result in the free energy of adsorption ${∆G}_{ads,i}$ for the respective probe molecule:

$${∆G}_{ads,i}=R∙T∙\ln\left(V_{G,i}\right)+C,$$ (3)

where R is the ideal gas constant and T is the absolute temperature. C is a constant depending on the reference state of adsorption. Since calculated differences or slopes from the ${∆G}_{ads,i}$ values are not affected by this constant, C is usually neglected for the determination of the free energy of adsorption.

The free adsorption energy can be split into two components contributing to the attractive forces of adsorption (Schrader and Loeb, 1992). These are the dispersive component of the adsorption energy ${∆G}_{ads}^{D}$ based on van der Waals interactions and the specific or polar component of the adsorption energy ${∆G}_{ads}^{D}$ based on electron pair donor–acceptor interactions. Both result in the sum of total free energy of adsorption ${∆G}_{ads}$ :

$${∆G}_{ads}={∆G}_{ads}^{D}+{∆G}_{ads}^{SP}.$$ (4)

In the following section, approaches are shown to determine these components individually from the total free energy of adsorption to determine physicochemical properties, such as free surface energy and acid–base properties.

#### 2.4.2 Method of topological index

The interpretation of the size of the free energies of adsorption ( ${∆G}_{ads}$ ) requires a characterization of the probe molecules with respect to their molecular properties such as vapor pressure (Saint Flour and Papirer, 1982), deformation polarizability (Donnet et al., 1991), and molecular geometry (Wiener, 1947).

One often applied approach was provided by Schultz et al. (1987) using surface tension data for characterization of the probe molecules according to their cross-sectional area a and their dispersive component of the surface energy $\gamma_{L}^{D}$ :

$$RTln\left(V_{G,i}\right)=f\left(a_{i}∙\sqrt{\gamma_{L,i}^{D}}\right).$$ (5)

Since the IGC measurements are performed under infinite dilution conditions, which implies that only the interaction of single molecules on a free surface is considered, the morphology and the properties of the surface under study have to be related to appropriate molecular features (other than the typical properties of bulk materials) of the probe molecules. Therefore, an accessible topological index was used, developed by Brendle and Papirer (1997a), which can be calculated from the structure of the probe molecules and is expressed as

$$RTln\left(V_{G,i}\right)=f\left(X_{T,i}\right).$$ (6)

The $X_{T}$ -value is related to the van der Waals volume of the molecules and, therefore, to the strength of dispersive interaction. For non-polar *n*-alkanes, $X_{T}$ corresponds to the number of carbon atoms. For polar probes, heteroatoms and bond types (Brendle and Papirer, 1997b) are additionally considered in the determination of $X_{T}$ .

The graphical presentation of the free energies of adsorption ( ${∆G}_{ads}$ ) against these structural parameters $X_{T}$ for all probes is required to separate dispersive ( ${∆G}_{ads}^{D}$ ) and polar components ( ${∆G}_{ads}^{SP})$ as follows.

Non-polar *n*-alkanes adsorb exclusively due to dispersive van der Waals interactions. Since the $X_{T}$ value in the case of *n*-alkanes corresponds to the number of carbon atoms (Brendle and Papirer, 1997a) and the free adsorption energy increases by the same increment as the chain length increases for each methyl group, a linear relationship can be observed for the alkanes. The so-called reference alkane line (compare Figure 3) can be used to determine the dispersive components of the adsorption energy ( ${∆G}_{ads}^{D}$ ) for polar probe molecules (Dorris and Gray, 1980). The difference between the total energy of adsorption $∆G_{ads,i}^{SP}$ and the dispersive component for the respective probe ( ${∆G}_{ads,i}^{D}$ ) yields the polar component ${∆G}_{ads,i}^{SP}$ , as can be seen in the following equation:

$$∆G_{ads,i}^{SP}={∆G}_{ads,i}-∆G_{ads,i}^{D}.$$ (7)

### 2.5 Quantum chemical simulation

A better understanding of physicochemical properties and interaction energies between probe molecules and a surface, as obtained by IGC, can be supported by molecular modeling using van der Waals and electrostatic interactions between atoms of the probe molecules and atoms of the adsorption sites. The simulation of larger surface structures also allows statements regarding the orientation of probes on the adsorbent and gives indications for bond lengths as well as bond types (Grimsey I. M. et al., 2002). Density functional theory (DFT) B3LYP hybrid functional (Lee et al., 1988; Becke, 1996) was used for quantum chemical calculations as implemented in Jaguar, version 10.3 (Schrodinger, 2019). As a model system for calculations, a 32 T (tetrahedral) silica cluster (cell size of approximately 1 nm × 1 nm) has been used in its non-hydroxylated and hydroxylated forms (no OH group and 2 OH groups/nm^2^, respectively), which resulted in a Si_32_O_68_H_8_ composition (108 atoms as a whole). The SiO_2_ cluster model and the adsorption structures of different probe molecules were optimized in the gas phase at the B3LYP/6-31G (d) level of theory, which seems to be a reliable method for studying the structures and stabilities of silica materials (Abdallah et al., 2009; Rimola et al., 2013). This computational model has also been successfully used in our previous works (Bauer et al., 2017; Bauer et al. 2019; Bauer et al. 2021). Test calculations on selected molecules were performed using the B3LYP/6–31 + G (d, p) approach showing the same trend. Overall, however, it must be noted that DFT calculations on such large systems are time consuming and, therefore, the use of Monte Carlo simulations may be a very promising approach (Kong et al., 2022). The energy of adsorption complex formation ( ${\Delta E}_{ads}$ ), which follows the same trend as the Gibbs free energy of reaction, was calculated at room temperature as the difference of electronic energies between the adsorption complex formed and its constituents when they are in their lowest energy state.

## 3 Results and discussion

### 3.1 Structural characterization of porous silica spheres

**FIGURE 1:**
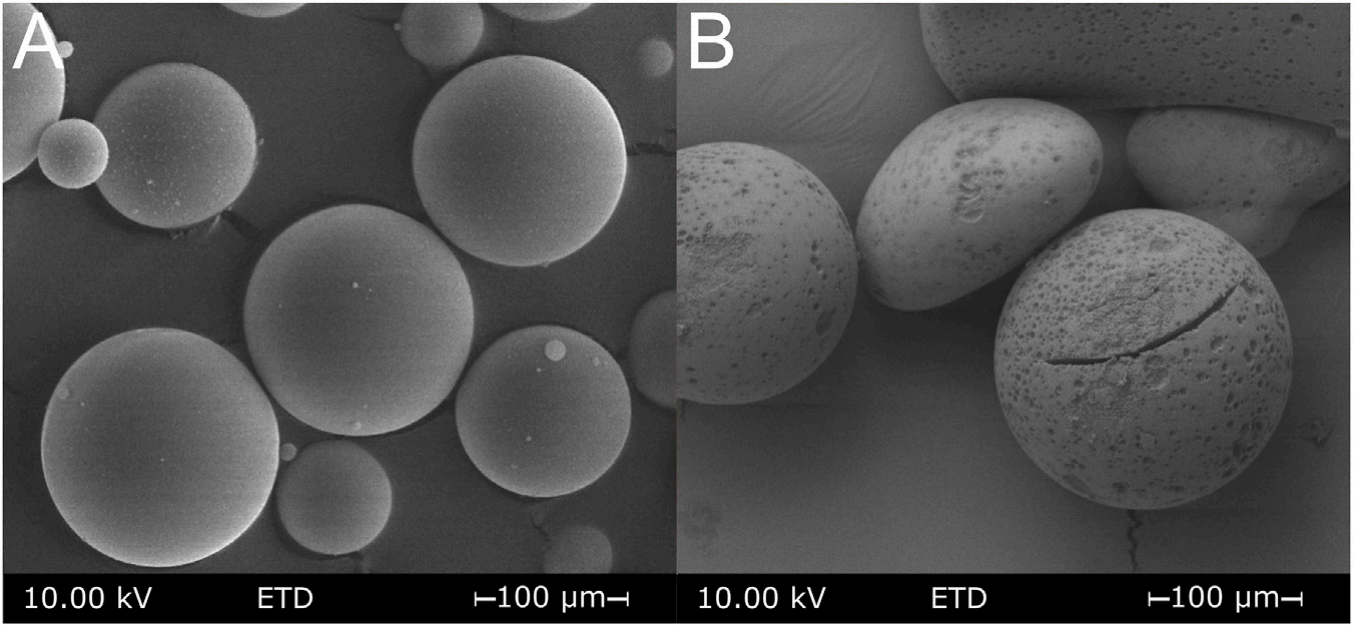
Electro-microscopic image of the silica spheres before **(A)** and after **(B)** the leaching procedure to create porosity.

The porous silica spheres produced were first examined by means of textural methods. Figure 1A shows an electro-microscopic image of the porous silica spheres. Thermally induced phase separation, followed by the removal of the outer skin and acid extraction of the borate-rich phase, produced a visible pore system (Figure 1B). The particles retain their spherical shape with a diameter of ∼100 µm. However, due to the alkaline extractions, distinct cavities can be observed on the spherical surface. A difference in texture due to surface modification cannot be detected by scanning electron microscopy on MPTMS-modified porous glass spheres. Particle size determination by laser diffraction showed a Gaussian particle size distribution in a range of 60–200 µm with an average value of 112.5 µm.

**FIGURE 2:**
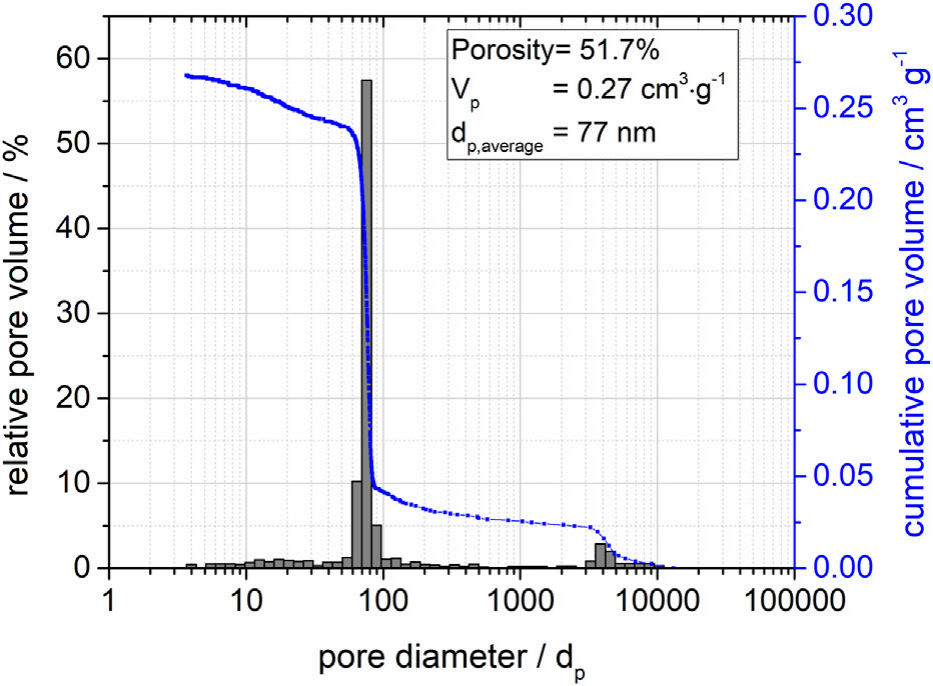
Pore distribution function for the porous silica spheres determined by mercury porosimetry showing relative and cumulative pore volumes.

According to the mercury porosimetry in Figure 2, the material exhibited a narrow mono-modal pore system. The average pore diameter is 77 nm at a porosity of 51.7% and a pore volume of 0.27 cm^2^/g. Signals of pore diameters at 4,000 nm can be attributed to an interparticulate pore volume, which occurs due to the packing of the silica spheres at a particle size of about 100 µm. As expected, no change in this pore structure due to post-synthetic silanization was observed.

The internal surface area of the pore system was determined by nitrogen sorption using BET theory. The specific BET surface area of the pure glass of 26 m^2^/g is reduced to 19 m^2^/g by silanization with MPTMS. Calculating the pore size distribution from desorption isotherms, a neglectable change in average pore diameter occurs for the modified glass. Therefore, the decrease of specific BET area after surface modification can be addressed to a reduction of surface roughness due to the grafting of MPTMS.

### 3.2 Determination of the free energy of adsorption

**FIGURE 3:**
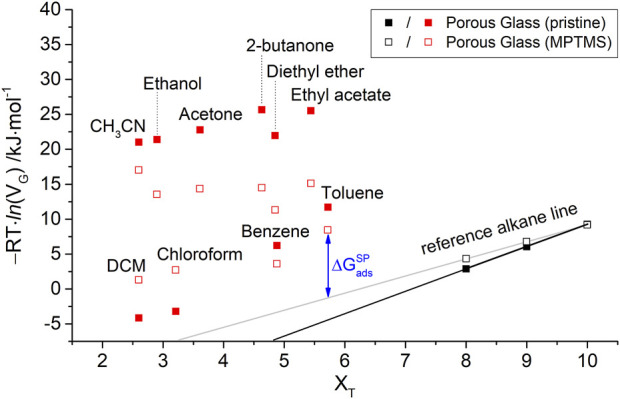
Free energy of adsorption $∆G_{ads}$ for *n*-alkanes and polar probe molecules on macro-porous silica before (■) and after (□) surface modification with MPTMS.

The free energy of adsorption ${∆G}_{ads}$ was estimated from the net retention time for pristine porous silica and MPTMS-modified silica within a temperature range from 30°C–120°C for *n*-alkanes and 90°C–120°C for polar probe molecules. The free energy of adsorption ${∆G}_{ads}$ as a function of the topological transcriptor $X_{T}$ is presented in Figure 3 for 120°C. The graph shows significant changes in the adsorption behavior for different probes on MPTMS-modified silica compared to pristine silica. Since the slope of the reference alkane line has decreased, a lower dispersive interaction is indicated for the silanized silica.

**FIGURE 4:**
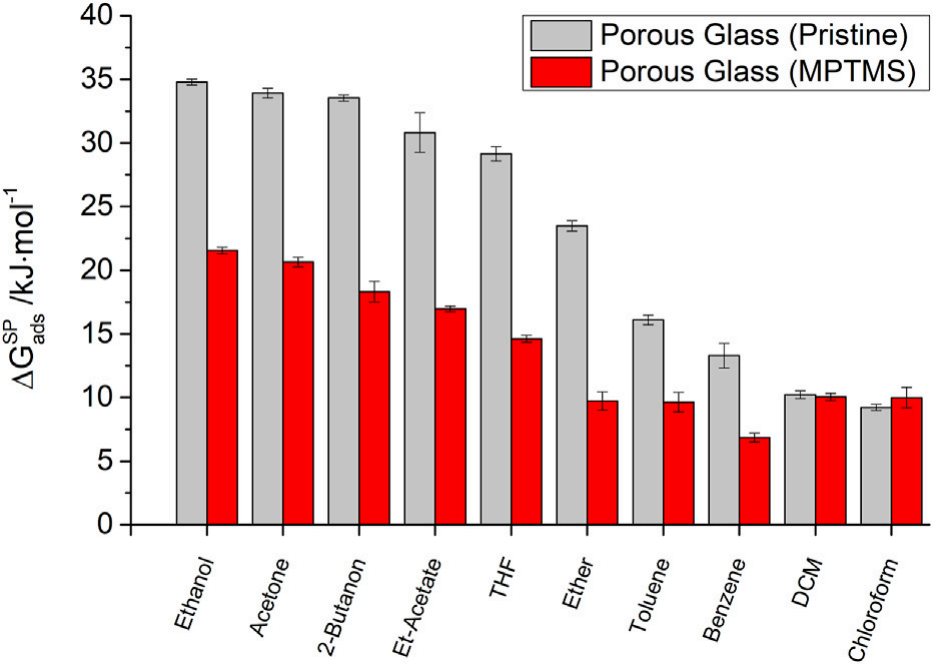
Polar component of the free energy of adsorption $∆G_{ads}^{SP}$ at 120°C for polar probe molecules on macro-porous silica before (gray) and after (red) surface modification with MPTMS.

For the evaluation of the interactions of polar probe molecules, separate consideration of the polar component of the adsorption free energy $∆G_{ads}^{SP}$ is crucial. To determine this value, as indicated in Figure 4 according to Eq. 7, the disperse component of the adsorption free energy ( $∆G_{ads}^{D}$ ) must be calculated by extrapolating the reference alkane line ( ral ), as shown in the following equation:

$$∆G_{ads,i}^{D}=s_{ral}∙X_{T,i}+n_{ral},$$ (8)

where $s_{ral}$ is the slope determined from the reference alkane line and $n_{ral}$ is the intercept. The results for $∆G_{ads,i}^{SP}$ are shown in Figure 4. For polar molecules, we can classify the $∆G_{ads,i}^{SP}$ and, therefore, the specific interaction strength by decreasing order:

Ethanol>Acetone>Butanone>Ethyl acetate>THF>Ether>Toluene≈Benzene≈DCM≈Chloroform.

This order already shows a correlation between the chemical structure of the polar probes and the strength of the observed specific interactions. The strongest interactions occur for probe molecules with the highest dielectric constants (ethanol and acetone) and, consequently, the ability to form hydrogen bonds. According to the classification, the formation of π-interactions for benzene or toluene plays a minor role in the polar interaction strength. Likewise, purely Lewis-acidic probe molecules such as chloroform (CHCl_3_) and dichloromethane (DCM) show only a weak interaction with the surface of the porous glass.

For MPTMS-modified silica samples in general, a decreasing polar component of the adsorption free energy ( $∆G_{ads}^{SP}$ ) is observed, indicating a weakening of polar interactions due to the modification. This is particularly true for polar probes that have oxygen-containing functional groups, such as alcohols, esters, and ketones. A sharp decrease in $∆G_{ads}^{SP}$ of polar probes due to modification is associated with the loss of free silanol groups in the course of MPTMS grafting. The $∆G_{ads}^{SP}$ values for chloroform and dichloromethane with 10.2 kJ/mol and 9.2 kJ/mol for pristine silica and 10.0 kJ/mol and 9.9 kJ/mol for the modified silica, respectively, remain almost unchanged.

This demonstrates that an insufficient number of polar probe molecules is inappropriate for IGC studies of polar surface sites. Only with a large spectrum of polar molecules is it possible to represent all the contributions to adsorptive–adsorbent interactions. The example of the mono-polar Lewis acid chloroform shows that this probe molecule is not sensitive to a loss of Lewis acid silanol groups due to the surface modification of porous glass. In contrast, Lewis basic probe molecules with the ability to form hydrogen bonds form particularly attractive interactions in the case of silanol groups and are, therefore, well suited for imaging the surface properties of porous glasses.

### 3.3 Determination of free surface energy

In accordance with the linear relationship found between the free energy of adsorption of *n*-alkanes and their chain length, Dorris and Gray (Dorris and Gray, 1980) provided a simple approach to calculate the dispersive contribution of the surface free energy ( $\gamma_{S}^{D}$ ). Using the data of *n*-alkanes in the IGC, the dispersive component of the free surface energy can be calculated according to the following equation:

$$\gamma_{S}^{D}=\frac{{\left({∆G}_{ads}^{\left({CH}_{2}\right)}\right)}^{2}}{4∙N_{A}^{2}∙{\left(a_{{CH}_{2}}\right)}^{2}∙\gamma_{\left({CH}_{2}\right)}^{D}},$$ (9)

where ( ${∆G}_{ads}^{\left({CH}_{2}\right)}$ ) is the CH_2_ increment of adsorption free energy in the reference alkane line, $N_{A}$ is the Avogadro constant, $a_{{CH}_{2}}$ is the cross sectional area of a CH_2_ group, and $\gamma_{\left({CH}_{2}\right)}^{D}$ is the surface energy of a theoretical polymer consisting of only methylene groups (Gaines, 1972).

**FIGURE 5:**
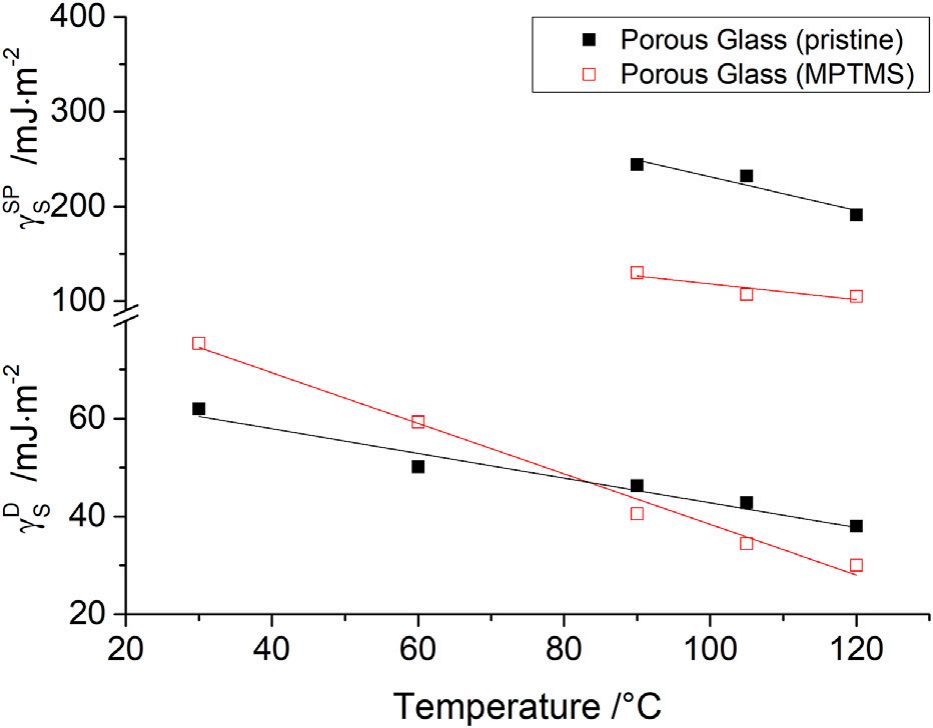
Dispersive $\gamma_{S}^{D}$ and specific $\gamma_{S}^{SP}$ components of the free surface energy as a function of temperature for macro-porous silica before (■) and (□) after surface modification with MPTMS.

For IGC measurement in the range from 30°C to 120°C, a temperature dependence of $\gamma_{S}^{D}$ for MPTMS modified and pristine silica is shown in Figure 5. For pristine silica, $\gamma_{S}^{D}$ values were found in a range from 38 to 63 mJ/m^2^ and for modified silica from 30 to 75 mJ/m^2^. In comparison, Rückriem et al. (2010) also investigated mesoporous silica and found dispersive free surface energy in the same magnitude with 40.7 mJ/m^2^ before and 50 mJ/m^2^ after surface modification with hexadimethylsilazane (HMDS) at 93°C. Pirez et al. (2014) similarly observed a steady increase in $\gamma_{S}^{D}$ from 34.1 mJ/m^2^ to 72.3 mJ/m^2^ for different degrees of surface modification of SBA-15 with organosilanes. On the contrary, a decrease of the dispersive surface energy due to silanization of amorphous precipitated silica from 86.7 mJ/m^2^ to 53.6 mJ/m^2^ (at 20°C) was observed by Castellano et al. (2012). Furthermore, Gamelas et al. (2018) also found a decrease in $\gamma_{S}^{D}$ values from 38 mJ/m^2^ to 14 mJ/m^2^ (at 40°C) for lignocellulosic fibers after modification with hydrophobic methylsilyl groups. However, Figure 5 shows from the temperature dependence of $\gamma_{S}^{D}$ that the interpretation of the change in dispersive surface energy due to modification at only one temperature is insufficient. In general, a decrease in $\gamma_{S}^{D}$ is observed at higher temperatures. Since this effect is more pronounced for modified glass, the $\gamma_{S}^{D}$ falls below that of pristine glass at temperatures above 85°C. Specifically, our IGC findings, exclusively using the adsorption data of *n*-alkanes, point out that MPTMS modification should yield, at high measuring temperatures, an even more hydrophilic silica surface, whereas $\gamma_{S}^{D}$ results obtained at room temperatures indicate the expected hydrophobization of silica.

As Bauer et al. have already shown in previous publications (Bauer et al., 2019; Bauer et al., 2021), the strength of purely disperse interactions on oxide materials is inferior to the strength of polar interactions. For this reason, surface characterization using only disperse components of the free surface energy $\gamma_{S}^{D}$ cannot be recommended.

In comparison with the determination of the dispersive component of the surface energy $\gamma_{s}^{d}$ *via* the slope of the *n*-alkane line, the estimation of the specific component of the surface energy $\gamma_{s}^{sp}$ is more complicated. Unfortunately, the values of ${∆G}_{ads}^{sp}$ do not result directly in the determination of the specific component of the surface energy ( $\gamma_{S}^{SP}$ ). Similar to the free enthalpy of adsorption, the free surface energy $\gamma_{S}^{total}$ is defined as a sum a dispersive component ( $\gamma_{S}^{D}$ ) and a polar component ( $\gamma_{S}^{SP}$ ) (van Oss, 2006):

$$\gamma_{S}^{total}=\gamma_{S}^{D}+\gamma_{S}^{SP}.$$ (10)

An adhesion approach postulated by van Oss et al. (1988) was used to describe the polar component of free surface energy $\gamma^{SP}$ with the help of electron pair donor and electron pair acceptor interactions between solid surface and polar probe molecules. Since these are corresponding donor–acceptor interactions, the parameter $\gamma^{SP}$ is further split into an electron acceptor (or Lewis acid) parameter ( $\gamma^{+}$ ) and an electron pair donor (or Lewis base) parameter ( $\gamma^{-}$ ).

During adsorption of a polar probe molecule (L) onto a solid surface (S), the interactions between the Lewis acid property of the probe molecules $\gamma_{L}^{+}$ and the Lewis base property of the solid $\gamma_{S}^{-}$ as well as the Lewis base property of the probe molecules $\gamma_{L}^{-}$ and the Lewis acid property of the solid $\gamma_{S}^{+}$ can be related to the polar free enthalpy of adsorption ${∆G}_{ads}^{SP}$ according to the following equation:

$$∆G_{ads,i}^{SP}=2∙a_{i}∙N_{A}∙\left(\sqrt{\gamma_{L}^{+}∙\gamma_{S}^{-}}+\sqrt{\gamma_{L}^{-}∙\gamma_{S}^{+}}\right),$$ (11)

where $N_{A}$ is the Avogadro constant and $a_{i}$ is the cross-sectional area of the individual probe molecule.

However, the van Oss approach is rarely applied in this original non-linear form. For the determination of $\gamma_{S}^{+}$ and $\gamma_{S}^{-}$ , pairs of mono-polar probes ( $\gamma_{L}^{+}=0$ or $\gamma_{L}^{-}=0$ ) are often used for calculation (Das et al., 2011; Mohammad, 2013) by eliminating one root term of Eq. 11 at a time.

Unfortunately, the obtained estimates for $\gamma_{S}^{+}$ and $\gamma_{S}^{-}$ depend on the pair of mono-polar probes molecules. However, such dependency of the polar component of the free surface energy $\gamma_{S}^{SP}$ on the chosen mono-polar probes molecules is especially not acceptable for highly polar silica materials, as shown by Bauer et al. (2019). To overcome this limitation and to obtain the best approximation for $\gamma_{S}^{+}$ and $\gamma_{S}^{-}$ parameters, a non-linear parameter estimation approach with simultaneous consideration of all polar probe molecules was used. This procedure was first recommended by Bauer et al. (2019) and corresponds to a weighted minimization of the error squares, as shown in the following equation:

$${\sum_{i}^{probes}\omega_{i}∙\left[∆G_{ads,i}^{SP}-2∙a_{i}∙N_{A}∙\left(\sqrt{\gamma_{L,i}^{+}∙\gamma_{S}^{-}}+\sqrt{\gamma_{L,i}^{-}∙\gamma_{S}^{+}}\right)\right]}^{2}=\mathrm{Min}!,$$ (12)

where $\omega_{i}$ is the weighting factor and $a_{i}$ is the cross-sectional area of the respective probe molecule i . The Lewis acid ( $\gamma_{L,i}^{+}$ ) and Lewis base parameters ( $\gamma_{L,i}^{-}$ ) for the individual probe molecules are given in the literature (van Oss, 2006).

The associated polar component of free surface energy $\gamma_{S}^{SP}$ was measured in a temperature range of 90°C–120°C due to stronger interactions of the polar probe molecules, as shown in Figure 5.

Values of $\gamma_{S}^{SP}$ for pristine silica were found in the range from 191 mJ/m^2^ to 244 mJ/m^2^ and they are, therefore, larger by a factor of six than the disperse component $\gamma_{S}^{D}$ at the corresponding temperature. Surface modification with MPTMS uniformly reduces $\gamma_{S}^{SP}$ for all temperatures investigated. Although this $\gamma_{S}^{SP}$ is significantly reduced, it still exceeds $\gamma_{S}^{D}$ by a factor of 2.5.

The reason for the decrease in polar interactions is the reduction of the surface silanol groups by silane modification. However, even after extensive silanization, the remaining polar silanol groups make an important contribution to the physicochemical properties of siliceous materials (Bauer et al., 2003; Bauer et al. 2019; Bauer et al. 2021).

After values for $\gamma_{S}^{+}$ and $\gamma_{S}^{+}$ have been determined for the surface of the solid, the $\gamma_{S}^{SP}$ can be calculated (van Oss et al., 1988) as shown in the following equation:

$$\gamma_{S}^{SP}=\sqrt{\gamma_{S}^{+}∙\gamma_{S}^{-}}.$$ (13)

As displayed in Table 1, the total amount of surface energy $\gamma_{S}^{total}$ is higher for the pristine glass than for the modified sample. It should be noted here that the measurement of polar probes at low temperatures was not technically possible. For this reason, the values of $\gamma_{S}^{SP}$ at 30°C and 60°C were approximated by extrapolation. Nevertheless, the grafting of MPTMS drastically lowers the surface energy $\gamma_{S}^{total}$ and, therefore, the interaction strength regardless of temperature.

**TABLE 1:** Estimated values for the free surface energy $\gamma_{S}^{total}$ and the dispersive $\gamma_{S}^{D}$ and polar $\gamma_{S}^{SP}$ components from 30°C to 120°C (*extrapolated values).

Temperature (°C)	$\gamma_{S}^{D}/mJ{∙m}^{-2}$		$\gamma_{S}^{SP}/mJ{∙m}^{-2}$		$\gamma_{S}^{total}/mJ{∙m}^{-2}$	
	Pristine porous silica	MPTMS-modified silica	Pristine porous silica	MPTMS-modified silica	Pristine porous silica	MPTMS-modified silica
30	62	75.4	355*	177*	417*	252.4*
60	50.1	59.3	302*	152*	352.1*	211.3*
90	46.3	40.5	244	130	290.3	170.5
105	42.8	34.4	232	107	274.8	141.4
120	38	30	191	105	229	135

### 3.4 Acid–base properties

In the field of adhesion, Fowkes (Fowkes, 1964; Fowkes, 1968) was the first who addressed the non-dispersive or specific interactions to acid–base or donor–acceptor interactions.

In the meantime, various models have been developed to investigate the acid–base properties by means of IGC. The Gutmann model (Gutmann, 1978) assigns parameters to the polar probe molecules of IGC according to their electron pair acceptor ability (AN) and electron pair donor ability (DN). Subsequently, acid ( $K_{A}$ ) and ( $K_{D}$ ) parameters can be calculated for the material under investigation according to the following equation:

$$∆H_{ads}^{SP}=AN∙K_{D}+DN∙K_{A}.$$ (14)

Plotting $∆H_{ads}^{SP}/AN$ against DN/AN yields in a linear relationship with slope $K_{A}$ . Unfortunately, the estimation of $K_{D}$ from the intercept may lead to a significant error (Shi et al., 2007). Therefore, $K_{D}$ is determined from the slope by the following relationship:

$$\frac{∆H_{ads}^{SP}}{DN}=\frac{AN}{DN}∙K_{D}+K_{A}.$$ (15)

However, the determination of acid–base properties from the thermodynamic parameter $∆H_{ads}^{SP}$ is very time consuming (Voelkel, 2012) since every probe must be measured for at least three different temperatures. As the enthalpy of adsorption and the free enthalpy of adsorption are proportional ( $∆H_{ads}^{SP}\propto ∆G_{ads}^{SP}$ ) (Mittal, 1991), an acceptable simplification (Voelkel, 1991; Voelkel et al., 2009) can be made by using $∆G_{ads}^{SP}$ for the Gutmann approach according to the following equation:

$$∆G_{ads}^{SP}\approx AN∙K_{D}+DN∙K_{A}.$$ (16)

Temperature dependency must be taken into account for these values, and this simplification is only valid if the entropic contributions to $∆G_{ads}^{SP}$ are negligibly small (Papirer et al., 1988).

Finally, the surface of the column material can be characterized in terms of Lewis acidity with the determined parameters $K_{A}$ and $K_{D}$ . $K_{A}$ describes the electron acceptor ability and $K_{D}$ the electron donor ability of the adsorption centers on the surface of the column material. Since it is not yet possible to designate a substance as acidic or basic from the results of the Gutmann approach, Lara and Schreiber (1991) suggest that a classification can be made from the $K_{A}{/K}_{D}$ ratio. Accordingly, acidic surfaces have $K_{A}{/K}_{D}$ > 1.1, basic surfaces $K_{A}{/K}_{D}$ < 0.9, and surfaces with 0.9 < $K_{A}{/K}_{D}$ < 1.1 are to be classified as amphoteric.

Two complications exist for the Gutmann approach with respect to IGC-ID, since the determination of AN and DN is performed in a probe molecule excess. First, many acidic probes also have basic sites and, therefore, tend to self-associate in solution (other than at infinite dilution). Second, at higher ratios of acid to base, 2:1 complexes may be formed preferentially (Riddle and Fowkes, 1990). This makes the interpretation and comparison of existing $K_{A}$ and $K_{D}$ values more difficult. Nevertheless, the Gutmann approach is an established method in the field of IGC, which has produced useful results for the surface characterization of solids.

Via Gutmann’s approach, for example, Sreekanth et al. (2018) assessed that the surface of melamine- and thiourea-derived graphitic carbon nitrides contain similar basic sites and fewer acidic sites. In the same way, Lazarević et al. (2011) proved that the iron-modification of sepiolite surface did not effect a change in acid–base properties, as the $K_{D}$ / $K_{A}$ ratio remained almost the same as that for natural sepiolite.

For materials such as graphitic carbon nitrides and sepiolite, which are not tested as frequently or completely as silica, what can be expected from the chemical intuition concerning any specific surface modifications? The reliable and effective handling of the various IGC techniques can provide some solid evidence rather than baseless suppositions.

In the literature, further enhancements of the Gutmann approach can be found, such as the corrected AN* values (Riddle and Fowkes, 1990) or an additional K-parameter for amphoteric contributions according to Hamieh et al. (2020).

**FIGURE 6:**
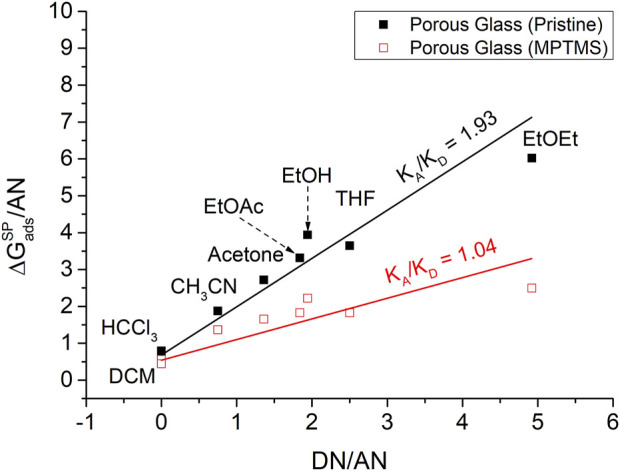
Determination of the acid and base parameters ( $K_{A}$ ; $K_{D}$ ) using the Gutmann approach at 120°C for macro-porous silica before (■) and after (□) surface modification with MPTMS.

$∆G_{ads}^{SP}$ values from Figure 4 were used to determine the acid–base properties on the modified and unmodified porous glass, as shown in Table 2. A decrease of the electron acceptor ability ( $K_{A}$ ) from 1.31 to 0.56 due to the surface modification and, thus, a decrease of the acidity of the silica sample are shown in Figure 6 for 120°C. The electron donor ability ( $K_{D}$ ) of the silica with values of 0.56 for pristine silica and 0.54 for modified silica remained almost unchanged. Overall, Gutmann’s results for the silica show a predominantly Lewis acidic character with $K_{A}/K_{D}$ of 1.93, which is significantly reduced by the surface modification to a value of $K_{A}/K_{D}$ of 1.04.

**TABLE 2:** Estimated acid–base parameters from the Gutmann and van Oss approachs at 120°C for porous silica before and after surface modification with MPTMS.

	$K_{A}$	$K_{D}$	$K_{A}/K_{D}$	$\gamma_{S}^{+}$	$\gamma_{S}^{-}$	$\gamma_{S}^{+}/\gamma_{S}^{-}$
Pristine porous silica	1.31	0.68	1.93	178 $mJ∙m^{-2}$	135 $mJ∙m^{-2}$	1.31
MPTMS-modified silica	0.56	0.54	1.04	51 $mJ∙m^{-2}$	143 $mJ∙m^{-2}$	0.36

As is well known, measuring surface energies of complex surfaces with non-polar probes only is worthless. A significant advantage of the van Oss approach (compared with the Gutmann approach) rests in the supply of the polar component of free surface energy $\gamma_{S}^{SP}$ , including its acid–base components. Hence, an alternative route for the determination of the acid–base properties results from the van Oss approach. Since $\gamma_{S}^{+}$ is a Lewis-acceptor parameter and $\gamma_{S}^{-}$ is a Lewis-donor parameter, they can both be related to each other to investigate the resulting electron donor–acceptor character ( $\gamma_{S}^{+}/\gamma_{S}^{-}$ ) for the studied material. A summary of the determination of $K_{A}$ , $K_{D}$ , and $K_{A}/K_{D}$ , as well as $\gamma_{S}^{+}$ , $\gamma_{S}^{-}$ , and $\gamma_{S}^{+}/\gamma_{S}^{-}$ , for pristine and MPTMS-modified porous glass can be found in Table 2. The determination of the van Oss values, which follows the theoretical approach of $\gamma_{L}$ parameters originated from surface tension experiments, nevertheless, underlines the results by Gutmann. The porous glass with $\gamma_{S}^{+}/\gamma_{S}^{-}$ of 1.32 can also be indicated as a Lewis acid, and by modification, the acidity decreases dramatically to a value of 0.36, leaving a Lewis-basic material.

The authors accept the argument of chemical intuition that removing −OH groups from silica makes its surface less polar. However, the van Oss approach provides numbers which give further information about the degree of reduction (see Figure 5).

In the IGC literature on controlled pristine porous glass, a Lewis-acidic character is mostly reported with $K_{A}/K_{D}$ = 2.13 (Rückriem et al., 2015), $K_{A}/K_{D}$ = 1.7 (Bauer et al., 2019), or even $K_{A}/K_{D}$ = 3.08 (Hamieh et al., 2001). The application of surfactants weakens this character in all cases. Typically, the silanol groups on the silica surface are used as binding sites. By covering these with modifying agents, electron donor–acceptor interactions are reduced, and adsorption inhibited, which is noticeable in the IGC measurement by the observed shortening of retention times.

### 3.5 Thermodynamic parameters and enthalpy–entropy compensation

The parameter of free energy of adsorption ${∆G}_{ads}$ depends not only on the respective probe molecule but also on the temperature at which it was determined. However, Greene and Past (1958) calculated the temperature-independent thermodynamic parameters such as adsorption enthalpy ( ${∆H}_{ads}$ ) and the adsorption entropy ( ${∆S}_{ads})$ from $RTln\left(V_{G}\right)$ at at least three different temperatures:

$$RTln\left(V_{G}\right)=∆H_{ads}-T∙∆S_{ads}.$$ (17)

Plotting $R∙\ln\left(V_{G}\right)$ against 1/T yields a straight line for every probe molecule with the slope of ${∆H}_{ads}$ and the intercept of ${∆S}_{ads}$ . The calculated thermodynamic parameters $∆H_{ads}$ and $∆S_{ads}$ determined from a temperature range of 30°C–120°C are presented for aliphatic probe molecules and for polar probe molecules in Table 3. As expected, the adsorption enthalpy $∆H_{ads}$ of longer alkyl chains increases due to stronger dispersive interactions caused by a higher polarizability of larger molecules. This effect is even more pronounced in the presence of mercaptopropyl groups of surface-modified porous glass. The increase in the polarizability of the surface due to the grafted MPTMS amplifies the dispersive van der Waals interactions and, thus, increases the adsorption enthalpy $∆H_{ads}$ of the alkane on the surface. Furthermore, as the chain length of the alkanes increases, an increase in the adsorption entropy $∆S_{ads}$ is also observed. The negative value ( $∆S_{ads}<0$ ) is due to the fact that as a result of the adsorption process, the probe molecules are localized on the solid surface and a higher order state is subsequently inherent in the system. Accordingly, the stronger interaction between the surface modification and the *n*-alkanes also leads to stronger localization and a more negative value for $∆S_{ads}$ .

**TABLE 3:** Estimated ${∆H}_{ads}$ and ${∆S}_{ads}$ values for non-polar and polar probe molecules on pristine and modified silica samples from 30°C up to 120°C.

Non-polar probes	Pristine porous silica		MPTMS-modified silica	
	$∆H_{ads}/kJ∙{mol}^{-1}$	$∆S_{ads}/J∙{mol}^{-1}∙K^{-1}$	$∆H_{ads}/kJ∙{mol}^{-1}$	$∆S_{ads}/J∙{mol}^{-1}∙K^{-1}$
Heptane	−50.3	−122.9	−45.3	−116.9
Octane	−56.9	−133.2	−51.6	−127.3
Nonane	−63.7	−144.1	−59.7	−142.6
Decane	−65.8	−142.8	−65.7	−152.5
Trimethylpentane	−50.4	−120.2	−43.2	−110.8
*c*-Octane	−55.5	−128.0	−55.5	−129.9
Acetonitrile	−55.3	−104.3	−63.6	−120.8
Acetone	−61.4	−108.8	−83.4	−175.0
THF	−64.8	−116.7	−84.4	−173.2
Ether	−69.5	−140.3	−101.5	−238.1
DCM	−44.8	−112.2	-	-
Chloroform	−49.1	−120.7	−43.8	−111.7
Ethyl acetate	−62.9	−110.5	−77.5	−163.3
Benzene	−59.5	−137.6	−53.8	−133.8
Toluene	−65.8	−144.6	-	-

In many thermodynamic analyses of chemical reactions and adsorption processes, it has been experimentally demonstrated that there are linear relationships between two thermodynamic or kinetic parameters in which the factors α and β are constant, as presented in the following equation:

$$∆H_{ads,i}=\alpha +\beta ∙∆S_{ads,i}.$$ (18)

This phenomenon is called enthalpy–entropy compensation (Liu and Guo, 2001) and can be used to address similar (or different) adsorption behavior of the probe molecules. Since the slope β of the regression line has the dimension of temperature, it is defined as the isokinetic temperature $T_{\beta }$ , at which the entire adsorption series should have the same rate (or equilibrium).

The observation of the compensation effect for widely different processes has led to a number of explanations, including ambiguous discussions (Yelon et al., 2012; Pan et al., 2015; Perez-Benito and Mulero-Raichs, 2016). It has also been proposed that the compensation effect is, in some cases, a result of trivial statistical errors and experimental uncertainties (Suárez et al., 1994). According to Krug et al. (1976a) and Krug et al. (1976b), the entropy–enthalpy compensation theory is only valid if the isokinetic temperature ( $T_{\beta }$ ) is not equal to the harmonic mean temperature ( $T_{hm}$ ) of the process under study.

Studies have shown that enthalpy–entropy compensation occurs not only during the adsorption of gases on active surfaces (Garrone et al., 2008; Korolev et al., 2011; Pera-Titus, 2016) but also in water–sorbent systems of dried foodstuff (Gabas et al., 1999; McMinn et al., 2005).

Originating from the enthalpy–entropy compensation, the isokinetic temperature is a useful value to distinguish between different adsorption/desorption mechanisms. For example, Pimentel and McClellan (1971) found different isokinetic temperatures for the formation of hydrogen bonds between ethers ( $T_{\beta }$ = 264°C), aldehydes ( $T_{\beta }$ = 49°C), ketones and esters ( $T_{\beta }$ = 185°C), amines ( $T_{\beta }$ = 171°C), and amides ( $T_{\beta }$ = 151°C) on phenol using enthalpy–entropy compensation.

Furthermore, the compensation effect between enthalpy and entropy reported for n-alkane sorption on different acidic zeolites (Eder and Lercher, 1997) reveals that the slopes, i.e., the isokinetic temperatures, are not equal; obviously due to differences in their sorption properties, which are affected by pore size, Si/Al ratio, etc. It should be noted that the difference in slopes can also be attributed to the existence of different surface sites (Díaz et al., 2007).

In the specific case of n-hexane adsorption on HZSM-5, Hercigonja et al. determined the compensation temperatures. Here, the lowest (−50°C) and the highest (69°C) isokinetic temperatures were found for parent HZMS-5 and for CuZSM-5, respectively. Importantly for the suitability of the isokinetic theory, all found compensation temperatures differ from the temperature of adsorption (30°C). These results clearly show that the highest changes in entropy of adsorbed n-hexane were achieved by its adsorption on the sample containing Cu^2+^ cations (Eder and Lercher, 1997).

**FIGURE 7:**
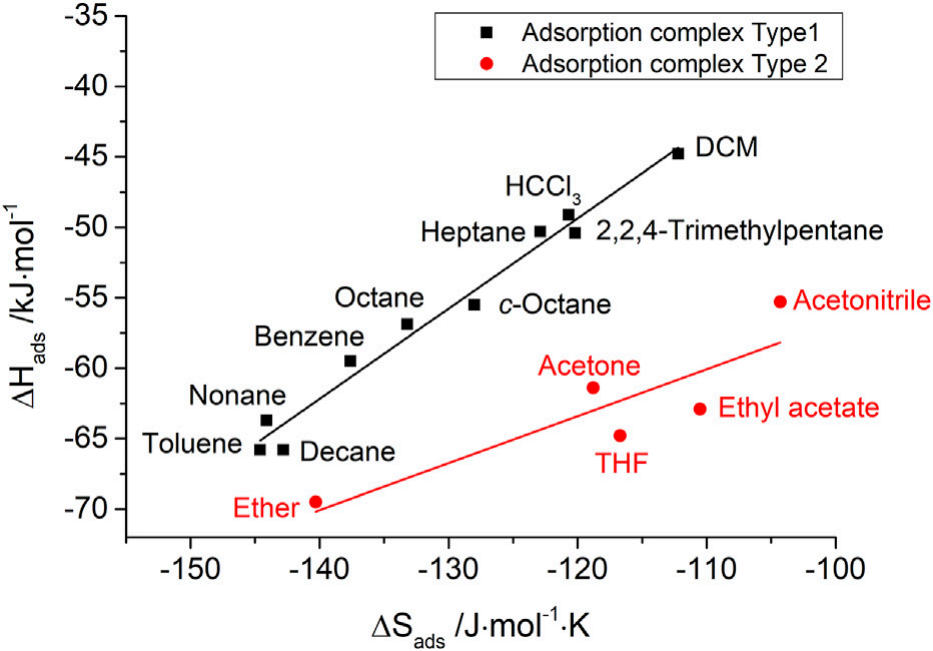
Enthalpy–entropy compensation for probe molecules with two types of adsorption complexes on pristine porous silica.

**FIGURE 8:**
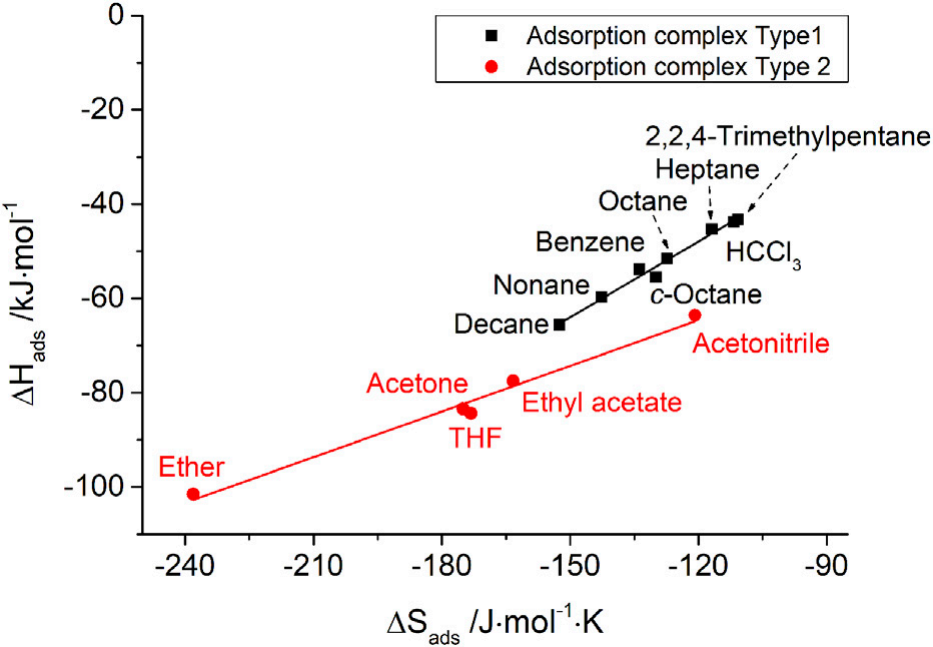
Enthalpy–entropy compensation for probe molecules with two types of adsorption complexes on MPTMS-modified porous silica.

The IGC data also show such enthalpy–entropy compensation effect for different kinds of probe molecules, as shown in Figures 7, 8 for the silica materials studied. Corresponding to the different isokinetic temperatures obtained from plotting $∆H_{ads}$ vs. $∆S_{ads}$ , two different adsorption complexes can be assumed to be present for both pristine and modified silica, as shown in Table 4. Linear *n*-alkanes, branched alkanes, and some polar probe molecules show a similar type of dependence with high isokinetic temperatures of 370°C for an adsorption on pristine silica. After surface modification, the isokinetic temperature is significantly reduced to 270°C, but it still strongly exceeds the experimental temperature $T_{m}$ of 105°C. This indicates that the adsorption is based on purely dispersive interactions. Interestingly, polar probe molecules with weak polar interactions (small $∆G_{ads}^{SP}$ ), such as chloroform, dichloromethane, benzene, and toluene, can also be assigned to the same adsorption complex (type 1) according to enthalpy–entropy compensation. From this, it follows that donor–acceptor interactions due to electron-rich heteroatoms of the chlorinated hydrocarbons and interactions due to electron π-systems of the aromatic compounds show a minor influence on adsorption and are exceeded by the van der Waals interactions during adsorption on the silica materials. In contrast, for polar probe molecules with strong polar interaction behavior (high $∆G_{ads}^{SP}$ ), such as diethyl ether, acetone, tetrahydrofuran, and ethyl acetate, a different adsorption complex (type 2) has to be assumed. For these adsorption complexes, low isokinetic temperatures of 60°C for pristine glass and 50°C for the surface-modified glass were found. All of the probe molecules that show strong polar interactions are able to form hydrogen bonds with the silica surface due to their oxygen- or nitrogen-containing functional groups.

**TABLE 4:** Two different assumed adsorption complexes (type 1 and type 2) and the related isokinetic temperatures for probe molecules on the silica surface according to enthalpy–entropy compensation before and after surface modification.

	Isokinetic temperature	
Adsorption complex	Pristine silica	MPTMS-modified silica
Type 1 (similar to aliphatic)	370°C	270°C
Type 2 (divergent from aliphatic)	60°C	50°C

The authors’ conclusion for the e–e compensation effect observed in this IGC study: on the basis of isokinetic temperatures, it has given reliable information about the very different nature of adsorption complexes of typically polar IGC probe molecules, e.g., chloroform (exclusively van der Waals interactions) and tetrahydrofuran (predominantly hydrogen bond formation). However, the scientific meaning of the height of isokinetic temperatures is still open to discussion.

### 3.6 Adsorption complexes and DFT investigations of polar probes on the silica surface

**FIGURE 9:**
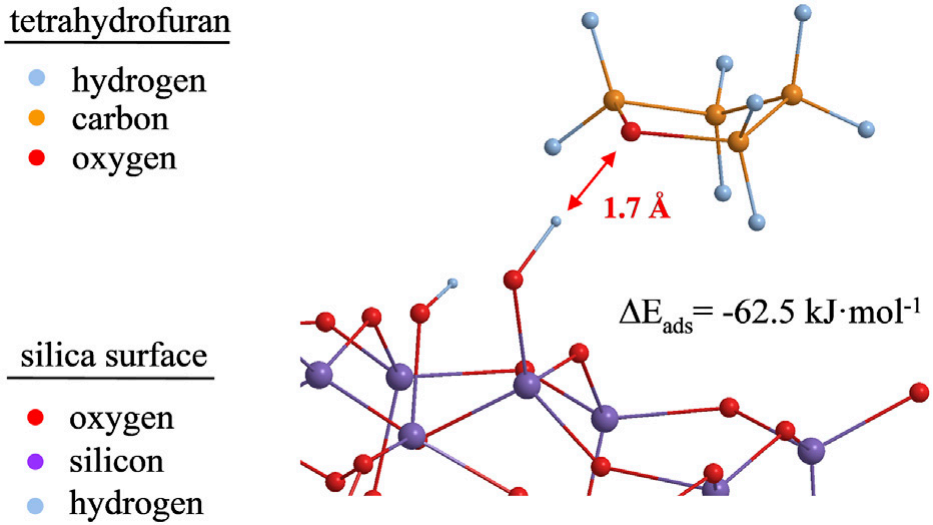
Optimized geometry for tetrahydrofuran adsorbed on silanol groups of a 32 Si-member silica cluster model at the B3LYP/6-31G (d,p) level of theory. For reasons of demonstrability, protons at the silica cluster’s boundaries are omitted.

DFT calculations on the adsorption of polar probes on the surface of a silica cluster model in its hydroxylated and non-hydroxylated form (2 OH groups/nm^2^ and no OH group, respectively) have been performed. Figure 9 shows an adsorbed tetrahydrofuran molecule in its optimized molecular structure at a distance of about 1.7 Å to one of the surface silanol groups.

**FIGURE 10:**
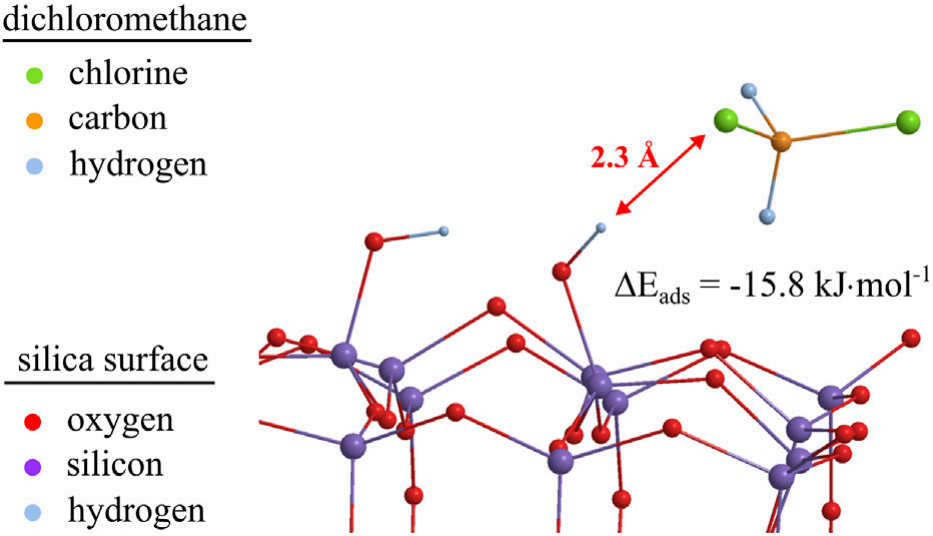
Optimized geometry for dichloromethane adsorbed on silanol groups of a 32 Si-member silica cluster model at the B3LYP/6-31G (d, p) level of theory. For reasons of demonstrability, protons at the silica cluster’s boundaries are omitted.

For comparison, Abdallah et al. (2009) found that, with the same B3LYP/6-31G(d) level of theory, bond lengths for hydrogen bonds were 1.7 Å and 1.9 Å for the physical adsorption of isopropanol on a silica surface. For THF adsorbed on the silica surface, not only the identical bond lengths, but also the localization of the Si–OH proton between the oxygen of the silanol group and the oxygen of the THF (Figure 9) indicate the formation of a hydrogen bond. The calculated energy of complexes formation ( $E_{ads}$ ) of THF adsorbed on silanol groups is approximately −62.5 kJ/mol. For comparison, an adsorbed dichloromethane molecule in its optimized molecular structure is visualized in Figure 10. The adsorption complex with a bond length of 2.3 Å and an $E_{ads}$ value of −15.8 kJ/mol prevents the formation of hydrogen bonds. In addition, the proton of Si–OH visibly does not participate in the bond formation with dichloromethane.

For comparison, free adsorption energies and bond distances for polar and non-polar probes on a neat silica cluster and a silica cluster with Si–OH groups are shown in Table 5. It should be noted that both non-polar and polar probe molecules show higher free adsorption energies when silanol groups are present on the surface of the silica cluster, pointing to stronger bonding of even dispersive interacting molecules with silanol groups. In comparison, the type 2adsorption complexes already show high adsorption energies ( $E_{ads}$ ) for the silanol-free silica cluster if the probe molecules have OH groups themselves. With silanol groups present on the surface, the highest adsorption energies can be found in the type 2 adsorption complexes. Furthermore, hydrogen bonds can be assumed for all calculated type 2 adsorption complexes according to the bond length of 1.7–1.9 A.

**TABLE 5:** Free adsorption energies and distances to the silica surface from DFT calculations for polar probe molecules on Si–OH free and Si–OH containing surfaces.

	Neat siloxane silica cluster		Silica cluster with Si–OH groups	
Probe molecule (adsorption complex)	Free adsorption energy $E_{ads}$ (kJ·mol^-1^)	Distance to the silica surface (Å)	Free adsorption energy $E_{ads}$ (kJ·mol^-1^	Distance to the silica surface (Å)
Adsorption type 1 (similar to aliphatic)				
n-Hexane	−3.1	2.9	−9.2	2.1
n-Octane	−1.2	3.1	−5.5	2.1
Benzene	−1.8	3.0	−30.6	2.3
Toluene	−0.8	3.6	−23.0	2.4
Dichloromethane	−1.9	2.3	−15.8	2.3
Adsorption type 2 (divergent to aliphatic)				
Tetrahydrofuran	−30.4	2.5	−62.5	1.7
Acetonitril	−9.4	3.0	−36.1	1.9
Iso-propanol	−50.2	2.0	−56.3	1.7

## 4 Conclusion

The technique of inverse gas chromatography (IGC) has been applied to investigate the effect of silanization on the physicochemical surface properties of siliceous materials. Thus, porous glass spheres with a mean particle diameter of 110 µm and a mean pore diameter of 77 nm were modified with a surface coverage of 6 MPTMS species/nm^2^ using 3-mercaptopropyltrimethoxysilane (MPTMS) as a grafting agent. The surfaces of the pristine and modified silica were analyzed by scanning electron microscopy (SEM), nitrogen sorption, mercury porosimetry, elemental and gravimetric analysis, and inverse gas chromatography in the infinite dilution mode, and particle size determination was performed using laser diffraction.

For both silica samples, the dispersive component of the surface energy ( $\gamma_{S}^{D}$ ) has been determined in the temperature range 30–120°C and was found to be 38 mJ/m^2^ on pristine porous glass and 30 mJ/m^2^ on the MPTMS-modified sample at an adsorption temperature of 120°C. The specific component of the surface energy ( $\gamma_{S}^{SP}$ ) has been obtained by measurements at 90–120°C *via* the van Oss approach and a least-squares procedure evaluating the IGC data of eight polar probe molecules collectively. As expected, all polar probes interact more strongly with the pristine surface ( $\gamma_{S}^{SP}$ = 191 mJ/m^2^ at 120°C) than with the MPTMS-modified sample surface ( $\gamma_{S}^{SP}$ = 105 mJ/m^2^) due to a noticeable loss of surface silanol groups through silanization. It should be noted that the polar component of the surface energy $\gamma_{S}^{SP}$ of porous silica exceeds the dispersive component $\gamma_{S}^{D}$ by a factor greater than 3 (even after silylation). These data show a reduction of the total free surface energy ( $\gamma_{S}^{total}$ ) from 229 mJ/m^2^ to 135 mJ/m^2^ after surface silylation, indicating both a reduced wettability and an increased hydrophobicity of the MPTMS-modified porous silica.

With respect to the acid–base surface properties determined according to the Gutmann as well as the van Oss approach, the Lewis-acidic parameters ( $K_{A}$ or $\gamma_{S}^{+}$ , respectively) exceed the Lewis-basic parameters ( $K_{D}$ or $\gamma_{S}^{-}$ , respectively) for porous siliceous glass. After the silanization process, the total acidity (i.e., the ratio $K_{A}/K_{D}$ or $\gamma_{S}^{+}/\gamma_{S}^{-}$ ) of the silica is reduced due to a loss of surface silanol groups, which results in an amphoteric (Gutmann) or even Lewis-basic (van Oss) silica surface.

According to the enthalpy–entropy compensation from the thermodynamic parameters, a correlation between ${∆H}_{ads}$ and ${∆S}_{ads}$ has been shown for both silica samples. Two types of adsorption complexes between the polar probe molecules and the silica surface are assumed because of different isokinetic temperatures. Type 1 adsorption complexes with an isokinetic temperature of 370°C were assigned to alkanes and weakly interacting polar probes such as benzene, toluene, dichloromethane, and chloroform. Strongly interacting polar probes, such as THF, diethyl ether, ethyl acetate, acetone, and acetonitrile, were assigned to another adsorption complex (type 2) and showed a significantly lower isokinetic temperature of 61°C on porous silica.

Quantum chemical studies on the adsorption of particularly polar probe molecules have proven useful in providing not only the surface configurations of the adsorbates but also in giving indications for the different adsorption complexes on the silica surface.

## Data Availability

The original contributions presented in the study are included in the article/Supplementary Material, further inquiries can be directed to the corresponding authors.
